# TopEnzyme: a framework and database for structural coverage of the functional enzyme space

**DOI:** 10.1093/bioinformatics/btad116

**Published:** 2023-03-08

**Authors:** Karel J van der Weg, Holger Gohlke

**Affiliations:** John von Neumann Institute for Computing (NIC), Jülich Supercomputing Centre (JSC), and Institute of Bio- and Geosciences (IBG-4: Bioinformatics), Forschungszentrum Jülich GmbH, Jülich 52425, Germany; John von Neumann Institute for Computing (NIC), Jülich Supercomputing Centre (JSC), and Institute of Bio- and Geosciences (IBG-4: Bioinformatics), Forschungszentrum Jülich GmbH, Jülich 52425, Germany; Institute for Pharmaceutical and Medicinal Chemistry, Heinrich Heine University Düsseldorf, Düsseldorf 40225, Germany

## Abstract

**Motivation:**

TopEnzyme is a database of structural enzyme models created with TopModel and is linked to the SWISS-MODEL repository and AlphaFold Protein Structure Database to provide an overview of structural coverage of the functional enzyme space for over 200 000 enzyme models. It allows the user to quickly obtain representative structural models for 60% of all known enzyme functions.

**Results:**

We assessed the models with TopScore and contributed 9039 good-quality and 1297 high-quality structures. Furthermore, we compared these models to AlphaFold2 models with TopScore and found that the TopScore differs only by 0.04 on average in favor of AlphaFold2. We tested TopModel and AlphaFold2 for targets not seen in the respective training databases and found that both methods create qualitatively similar structures. When no experimental structures are available, this database will facilitate quick access to structural models across the currently most extensive structural coverage of the functional enzyme space within Swiss-Prot.

**Availability and implementation:**

We provide a full web interface to the database at https://cpclab.uni-duesseldorf.de/topenzyme/.

## 1 Background

Recent developments in high-throughput sequencing methods led to a massive increase in sequence information. Databases such as the UniProtKB (The UniProt Consortium 2021) contain over 225 000 000 sequence records, of which over 550 000 are manually annotated and reviewed in Swiss-Prot. In contrast, the Protein Data Bank (PDB) (Berman et al*.* 2003), the worldwide repository of information about the 3D structure of biomolecules, contained 185 539 crystal structures at the end of 2021, of which many are redundant structures, and not all are enzymes. Generally, the topology (fold) of an enzyme is thought to be the major determinant for the given function (Hegyi and Gerstein 1999; Orengo et al. 1999). Currently, enzyme function prediction methods often use structures from the PDB for the training data set. However, this can lead to biases in prediction from the protein topology, especially for proteins with a similar topology but a different function, such as TIM-barrels (Nagano et al. 2002) and Rossman folds (Medvedev et al. 2019). With recent improvements in protein structure prediction methods (Mulnaes and Gohlke 2018; Mulnaes et al. 2020; Baek et al. 2021; Jumper et al. 2021), the availability of high-quality structural models has increased (Varadi et al. 2022). Such structural models will contribute to better coverage and balance of the structural enzyme space.

Currently used databases that categorize structural relationships are, among others, SCOP2 (Andreeva et al. 2014) (Structural Classification of Proteins), CATH (Sillitoe et al. 2021) (CATH Protein Structure Classification Database), and ECOD (Cheng et al. 2014) (Evolutionary Classification of Protein Domains). These databases provide a detailed and comprehensive description of the structural evolutionary relationships between proteins whose 3D structure has been deposited in the PDB.

Although these databases provide information on the structural characteristics of the protein and the related functions, further analyses using database-specific classifiers are required to obtain the structural information related to the function. While CATH provides FuncFams (functional families), these are not based on the enzymatic functions as classified by the Nomenclature Committee of the International Union of Biochemistry and Molecular Biology (IUBMB) (https://iubmb.org/). The IUBMB currently curates the list of enzyme commission (EC) numbers. To our knowledge, only the Enzyme Structure Database (ESD) and IntEnz databases from the EMBL-EBI relate structures to the enzyme classification. However, the ESD has not been updated since 2018 and does not cover the newest enzyme class, translocases. The IntEnz database contains no structural information on translocases, as it is based on the nonupdated ESD. Furthermore, both databases do not contain any structural information obtained from modeling sources such as the AlphaFold Protein Structure Database (Varadi et al. 2022) (AlphaFold DB) or the SWISS-MODEL repository (Bienert et al. 2017).

In this study, we introduce the database TopEnzyme, where 15 500 representative structural models are categorized by enzyme classification numbers for the currently largest structural coverage of functional enzyme space in the UniprotKB/Swiss-Prot. TopEnzyme, which includes additional information from SWISS-MODEL repository and the AlphaFold DB, provides a comprehensive overview and facilitates access to obtain structures associated with specific enzyme functions.

When we started the generation of TopEnzyme, only 22% of the structural enzyme space was covered with respect to the available sequence information in the PDB. Using our deep learning- and template-based software TopModel (Mulnaes et al. 2020), we generated structural models of 10 125 enzyme domains covering 4758 different folds, increasing the coverage to 35%. With the release of AlphaFold2 (Jumper et al. 2021) and its goal to model the full UniprotKB/Swiss-Prot (Varadi et al. 2022), the current structural coverage of the functional enzyme space is at 60% across the SWISS-Model repository, TopEnzyme, and AlphaFold DB, covering all available sequences with EC annotation in the manually curated UniProtKB/Swiss-Prot. The recent release of AlphaFold2 structures for the unreviewed UniprotKB/TrEMBL are not included in this investigation. We made use of the availability of two complementary protein structure prediction methods to mutually validate structural models and provide a comparison of the structural quality for 2419 models.

## 2 Construction and content

Using ExpasyEnzyme (Bairoch 2000) (accessed on 12 May 2022), we obtained a complete list of UniprotAC identifiers for 241 125 sequences with an enzyme function annotation according to EC numbers from Swiss-Prot. The first three levels of EC numbers represent the main-, sub-, and subsub-class functions, while the fourth level is the specific enzyme function designation. For example, the small monomeric GTPase with designation 3.6.5.2 is a hydrolase (3) that acts on acid anhydrides (6) and specifically on guanosine triphosphate (GTP) to facilitate cellular and subcellular movements (5). In total, we find 252 subsub-classes spanning 4926 unique designations with available sequence data. For many unique designations, sequence information is not available in the UniprotKB/Swiss-Prot. Using MMseqs2 (Steinegger and Söding 2017), we clustered the obtained sequences with an identity cut-off of 30%, such that each cluster represents a homologous cluster in the enzyme fold space (Rost 1999; Koehl and Levitt 2002; Pearson 2013). When a cluster contains more than one subsub-class, we split this cluster into smaller clusters such that we identify a representative for each subsub-class function. For each cluster, we aimed at modeling the representative with TopModel, using templates with a sequence identity > 30% and a sequence coverage > 80% (Data S1). The refinement procedure in TopModel was skipped as the strict restraints set by the chosen templates should provide structures of sufficient quality while keeping computational costs to a feasible level. This is confirmed for 70 structural models, 10 randomly selected from each enzyme mainclass, which were evaluated as to the effect the refinement procedure has on the TopScore (Mulnaes and Gohlke 2018) of such models compared to ones without refinement; TopScore is a meta Model Quality Assessment Program (MQAP) using deep neural networks to combine scores from 15 different primary predictors to predict the quality of protein structural models and highly correlates to the local superposition-free score lDDT (Mariani et al. 2013). The refinement improved the models by a TopScore of only 0.06 on average (Supplementary Fig. S1). TopModel allows for manual template selection, e.g. offering the ability to use templates with bound ligands. However, by restricting the possible pool of templates, this might limit the availability of templates with sufficient quality. Thus, we opted to use all available templates to create more enzyme structural models.

In the case of multi-domain enzymes for which template information is missing for one or more noncatalytic domains, we remove unmodeled regions according to the following criteria: (i) Must be at least ten residues long. (ii) Must not contain residues of secondary structure elements (α-helix or β-strand) longer than five residues. (iii) Must have a median relative solvent accessible surface area larger than 0.40. (iv) Must have a median contact density smaller than four contacts. This prevents the removal of loops close to the binding site. (v) We only remove unmodeled regions from the C- or N-terminal to keep loops within the modeled domain(s). In the case of multi-domain enzymes for which the template contains information on multiple domains, we often model the complete structure.

**Figure 1:**
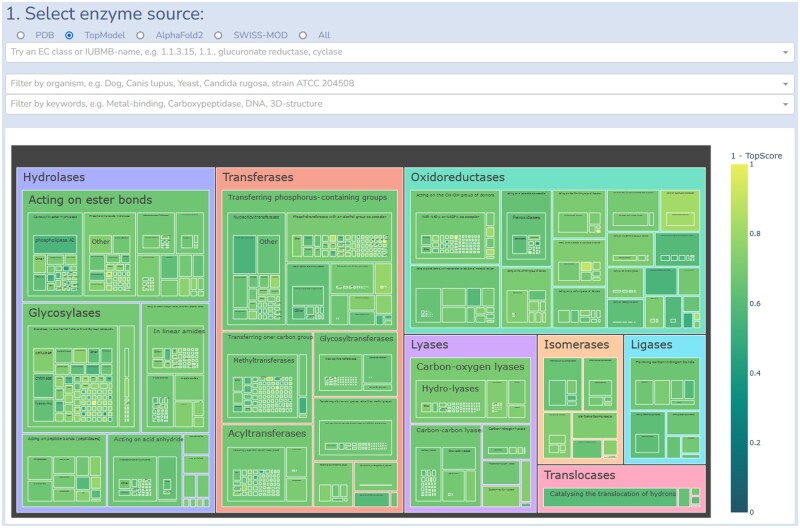
Enzyme map presenting the coverage of EC space with structural models. A screenshot of the interactively explorable enzyme map available at https://cpclab.uni-duesseldorf.de/topenzyme/. The color represents the structural scoring for representative models obtained from each database. The rectangle size represents the number of representative structures for the specific function. The treemap is ordered according to EC classification. By clicking on an area, the next subclass enlarges and shows the information for enzymatic function. The user can select between different database sources, PDB, TopModel, SWISS-MODEL repository, and AlphaFold2 for model selection. A search bar for EC classes and IUBMB names is provided, along with a filter for organisms and keywords. Tables below the treemap display all available UniprotAC representatives with EC numbers and a link to the PDB, TopModel, and SWISS-MODEL repository, and AlphaFold2 models. Clicking the UniprotAC identifier in the representative table opens the member table and shows all available models for cluster members. By clicking on the UniprotAC identifier in the member table, the information panel is opened, which contains links to the Uniprot, ExplorEnz, KEGG, BRENDA, and Expasy databases. Furthermore, a summary of the function with experimental evidence identifiers is given. We show the organism and strain from which the sequence is obtained and keywords for finding similar enzymes within the database

We created an interactive treemap of the resulting EC space and associated structural models of enzymes (Fig. 1, https://cpclab.uni-duesseldorf.de/topenzyme/). Each section is mapped by the main-, sub-, and subsub-class, as well as designation EC labels. The size of each section represents the proportion of the EC space as given by the number of representative sequences with this EC number. The color represents the average score of the structural representatives: Depending on the model source selected, we provide the pLDDT score (Jumper et al. 2021), a confidence measure of predicted structural quality for models from AlphaFold2, 1 - TopScore, a meta-MQAP predicting structural quality for models from TopModel, and QMEAN6, a linear combination of six statistical potential terms, related to the Z-score of X-ray structures (Benkert et al. 2011) for models from the SWISS-MODEL repository. In all three cases, values close to 1 indicate high-quality structures and values close to 0 the opposite. Above the treemap, we provide a search functionality for EC numbers and IUBMB names together with two filters for organisms and keywords. A representative table below the treemap is updated based on the search, filter, and map navigation input of the user. Here, the representatives for the current selection are shown with known EC numbers and links to the PDB, AlphaFold2, TopModel, and SWISS-MODEL models. By clicking on the UniprotAC identifier of the representative, a member table opens containing the same information for members in each representative cluster. By clicking on the UniprotAC identifier of the member, an information tablet opens containing links to the UniProt, ExplorEnz, KEGG, Brenda, and Expasy databases. Furthermore, a summary of the function with experimental evidence identifiers is given. We show the organism and strain from which the sequence is obtained and keywords for finding similar enzymes within the database. We also added a home page describing the user interface and a contact page for any inquiries or questions regarding the TopEnzyme database. The meta-data required to create this treemap is available as a csv file in the Supporting Information Data S2.

**Figure 2:**
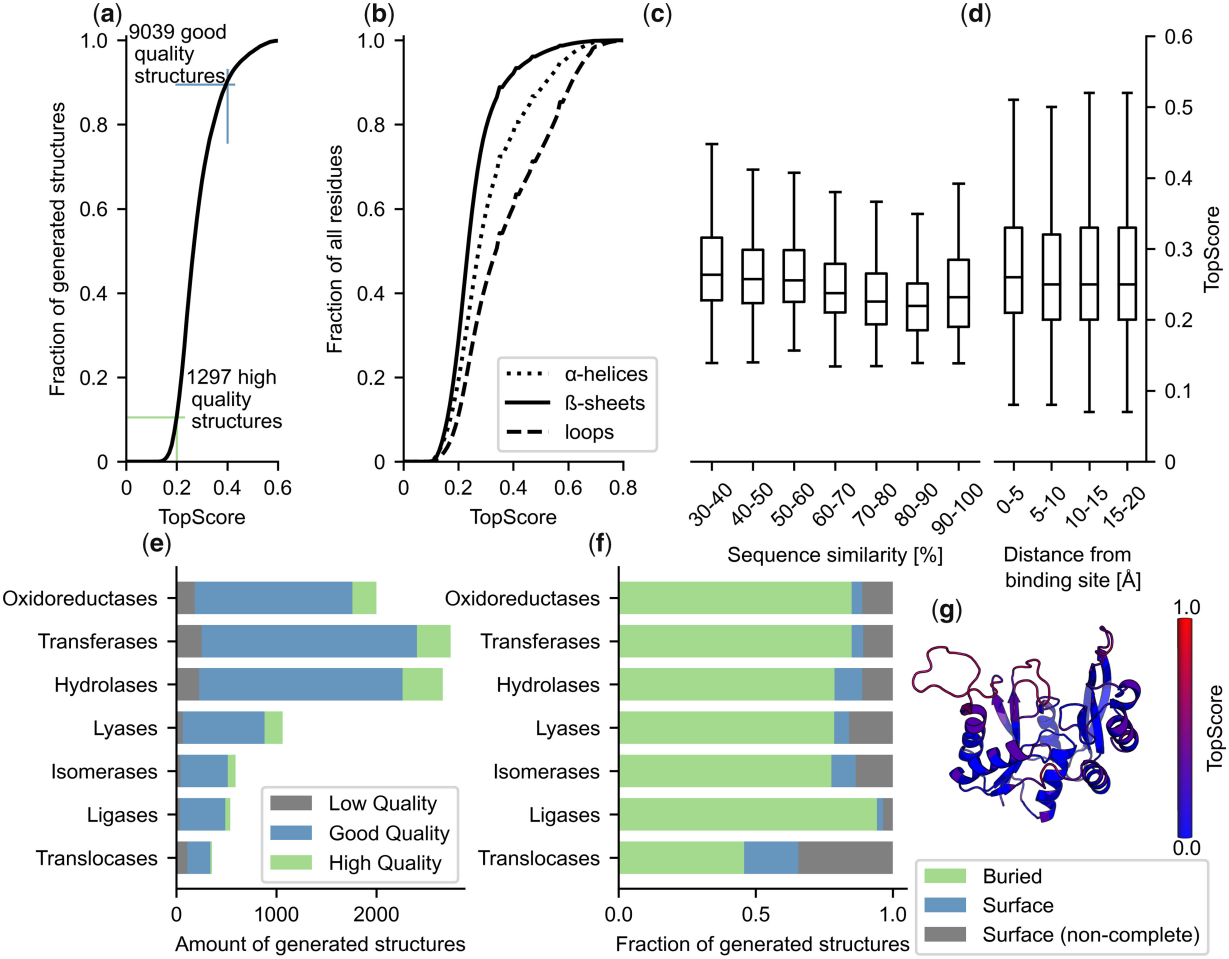
Quality assessment of enzyme models generated with TopModel. (a) TopScore for all (*n* = 9947) models predicted with TopModel. All models are generated with a template identity > 30% and a coverage > 80%. The lines indicate the cut-offs for TopScore values associated with high quality (TopScore < 0.2; *n* = 1297) and good quality (TopScore < 0.4; *n* = 9039) structures. TopScore values are bounded between 0 and 1; a lower TopScore is better. (b) Residue-wise TopScore for all model residues (*n* = 3 191 133) grouped by structural features. The continuous line represents the β-sheet residues (*n* = 629 778), the dash-dot line represents the α-helix residues (*n* = 1 961 309), and the dotted line represents the loop residues (*n* = 600 039). (c) The distribution of model quality based on the sequence similarity to the template used. The whiskers show the full range of TopScore values. The median is shown on the horizontal notch. (d) TopScore values for all (*n* = 3 191 133) model residues. The scores are clustered by the distance from the binding site. The boxplot properties are the same as in c. (e) Coverage of the generated structures by the main enzyme class. The horizontal bars are separated by low (TopScore ≥ 0.4), good (0.2 ≤ TopScore < 0.4), and high (TopScore < 0.2) quality structures. (f) Fraction of generated structures categorized by the main enzyme class with binding interfaces within a domain (‘buried’), binding interfaces on the surface of a domain with (‘surface’) and without [‘surface (noncomplete)’] known complementary domain(s). (g) An example structure (UniprotAC: Q9CQ28) highlighting the structural per-residue quality as judged by TopScore (see color scale; lower is better). The image is generated using PyMOL 2.3.0 (PyMOL)

Using TopScore, we analyzed the quality of the predicted models. Ninety percent of these models are of good quality (TopScore < 0.4), equivalent to 9039 structures spanning 233 subsub-classes (Fig. 2a and e). As to secondary structures, TopModel works better for predicting α-helices and β-sheets than loop conformations (Fig. 2b). This effect might be caused by bypassing the refinement stage (Supplementary Fig. S1). As expected, the score of our models increases with the sequence similarity of the template, except for models with a sequence similarity > 90% (Fig. 2c). Overall, the structural quality of the binding site is similar to that of the entire enzyme. However, we see a larger spread in per-residue scores around the binding site (Fig. 2d): Often, secondary structure features in the binding site are of high quality, whereas loop regions contribute to the lower-scoring residues. The exact structure of these loop regions may be less relevant to characterizing the dynamic nature of the protein binding site as these loops have often been shown to move to accommodate the space required for binding the ligand. The spread in binding site model quality might also be due to differences in the structural completeness of the binding sites, e.g. in the case of multidomain enzymes or allosteric enzymes where activation depends on domain- and ligand binding interactions. Using PocketAnalyzerPCA (Craig et al. 2011), we determined the average degree of buriedness (DOB) of our binding sites. The majority (77%) of the binding sites are characterized as inside a domain and, hence, are considered complete (Fig. 2f). For the binding sites on the surface, we categorize two types: surface and surface (noncomplete). The latter fraction (15% of all binding sites) is determined by mapping the binding site location to the homologous template and identifying the presence of not modeled complementary surfaces from global stoichiometry information in the template. In contrast, for the surface fraction (8%), we could not find structural information for complementary interfaces. This does not mean that there is no complementary surface present, just that there is no available information. Each structural model contains the residue-wise TopScore in the B-factor column of the PDB file. This allows the user to investigate the model confidence for specific regions (Fig. 2g).

## 3 Utility and discussion

The intended use of the database is to facilitate research connecting enzyme structure and biochemical function. The database serves this aim with its framework of covering EC space with structural models and easy applicability for users with different levels of expertise (see below). By using familiar identifiers, UniprotACs and EC numbers, and linking to other databases, such as the AlphaFold DB and SWISS-MODEL repository as well as ExploreEnz, KEGG, BRENDA and Expasy, we provide a framework for comprehensive structural enzyme information linked to enzymatic function.

We envision using generated models over crystal structures important for prediction methods for several reasons. First, some structural noise could make the machine learning method more robust to uncertain information (Mahajan et al. 2018; Plappert et al. 2018). Second, proteins are not rigid objects; having a uniform way of generating structural models should be advantageous compared to using experimental structures from different sources or binding states. Third, databases of predicted structural models cover a larger functional enzyme space. Last, this allows for extendibility to information from, e.g. metagenomic approaches, where no structural information is available, but sequences are deposited in the UniProtKB.

Compared to current databases such as the SCOP2 and CATH, our focus is on enzymatic function linked to available enzyme structural models. TopEnzyme starts from enzyme function categorization and provides available structural models from the respective fold space with easy access from the largest collection of generated enzyme models. There are two methods to interact with the database: (i) For scientists interested in large-scale analysis, we provide a csv file containing all the meta-data for each UniprotAC identifier. This allows users to download the latest release and incorporate the information into their workflows. (ii) For scientists interested in a few cases with specific enzyme functions, we provide a visualization in the form of the treemap (Fig. 1) hosted on https://cpclab.uni-duesseldorf.de/topenzyme/. The treemap allows users to browse enzymes with specific functions and provides a simple download method to obtain the representative models from the linked databases. In our own project, we used TopEnzyme to quickly obtain representative enzyme structures for building large datasets for a deep learning-based EC number classification.

We plan to update TopEnzyme when there is a new major release to databases of enzyme structural models or structural information for previously unlinked EC classes. As to new features, we intend to integrate a more exhaustive structural data collection from linked databases, as, currently, we collect only the best-ranked structural model from each method, while some methods produce ensembles of models. Furthermore, we will improve the search options to include a list of enzymatic functions to move the treemap to the selected function and include structural visualization when selecting a treemap node.

## 4 Comparison to AlphaFold2

**Figure 3:**
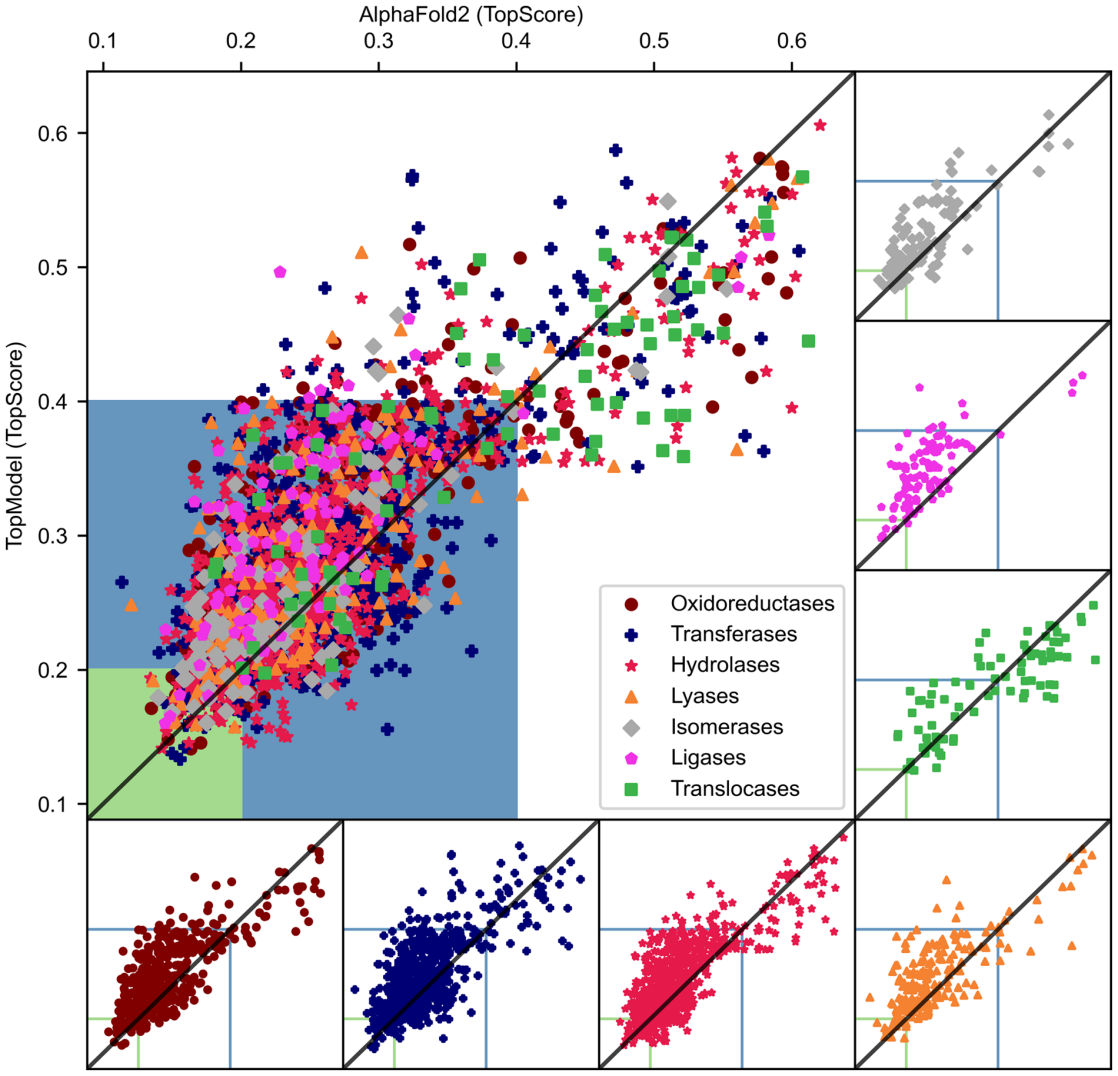
Comparison of structural models generated by TopModel and AlphaFold2. The models generated by TopModel or AlphaFold2 were scored with TopScore. For TopModel, all models are generated with a sequence similarity of > 30% and a coverage of > 80% with respect to the target. The AlphaFold2 models are obtained from the AlphaFold DB, alphafold.ebi.ac.uk. We compared 20% of the models generated by TopModel in the good (TopScore < 0.4; *n* = 1935) and 5% of the models in the bad (TopScore ≥ 0.4; *n* = 484) regime for each enzyme main class against AlphaFold2 structures. The diagonal line represents an equal score between the models. Data points above the diagonal favor AlphaFold2 structures, and data points below the diagonal favor TopModel structures. The blue area (TopScore ⩽ 0.4) represents the score for good-quality models, and the green area (TopScore ⩽ 0.2) represents the score for high-quality models. The panels around the figure show the same content but separated by enzyme main class

To obtain further insights into the quality of our structural models, we compare a proportion of our enzyme structural models with AlphaFold2 (Jumper et al. 2021) structures from the AlphaFold DB for the same enzymes. We remove disordered domains on the AlphaFold2 structures in the same way as in ours for fairness. The current AlphaFold2 implementation only folds single domains, although other implementations based on AlphaFold2 have been described that can predict multimers (Evans et al. 2021; Mirdita et al. 2021). We randomly selected 25% in each main class of our database for comparison to AlphaFold2 (Fig. 3). While the majority of TopEnzyme structures are available in the AlphaFold DB, TopEnzyme contains few structures unmodeled in the AlphaFold DB yet. We compared TopScore to pLDDT for both regimes (Supplementary Fig. S2). As TopScore and pLDDT predict (1—lDDT) (Mulnaes and Gohlke 2018) and lDDT (Jumper et al. 2021), both correlate significantly and fairly (*P* < .001, *R*^2^ = 0.59). Remarkably, when comparing computational AlphaFold2 models to experimental enzyme structures unseen by both methods (Supplementary Fig. S3), TopScore underestimates and pLDDT overestimates the true lDDT against the X-ray structure. We investigated the majority in the good-quality regime (TopScore values < 0.4; *n* = 1935) and a smaller number in the poor-quality regime (TopScore values ≥ 0.4; *n* = 484) to obtain a comprehensive overview. In the good-qualitative regime, AlphaFold2 performs slightly better than TopModel as judged by TopScore values computed for each model pair, which is consistent across all enzyme main classes. However, in the poor-quality regime, TopModel creates better models than AlphaFold2 for most enzyme classes except transferases and hydrolases. This result suggests that for some target sequences a model created from one or more homologous templates might be better.

## 5 Comparison to experimental structures

**Figure 4:**
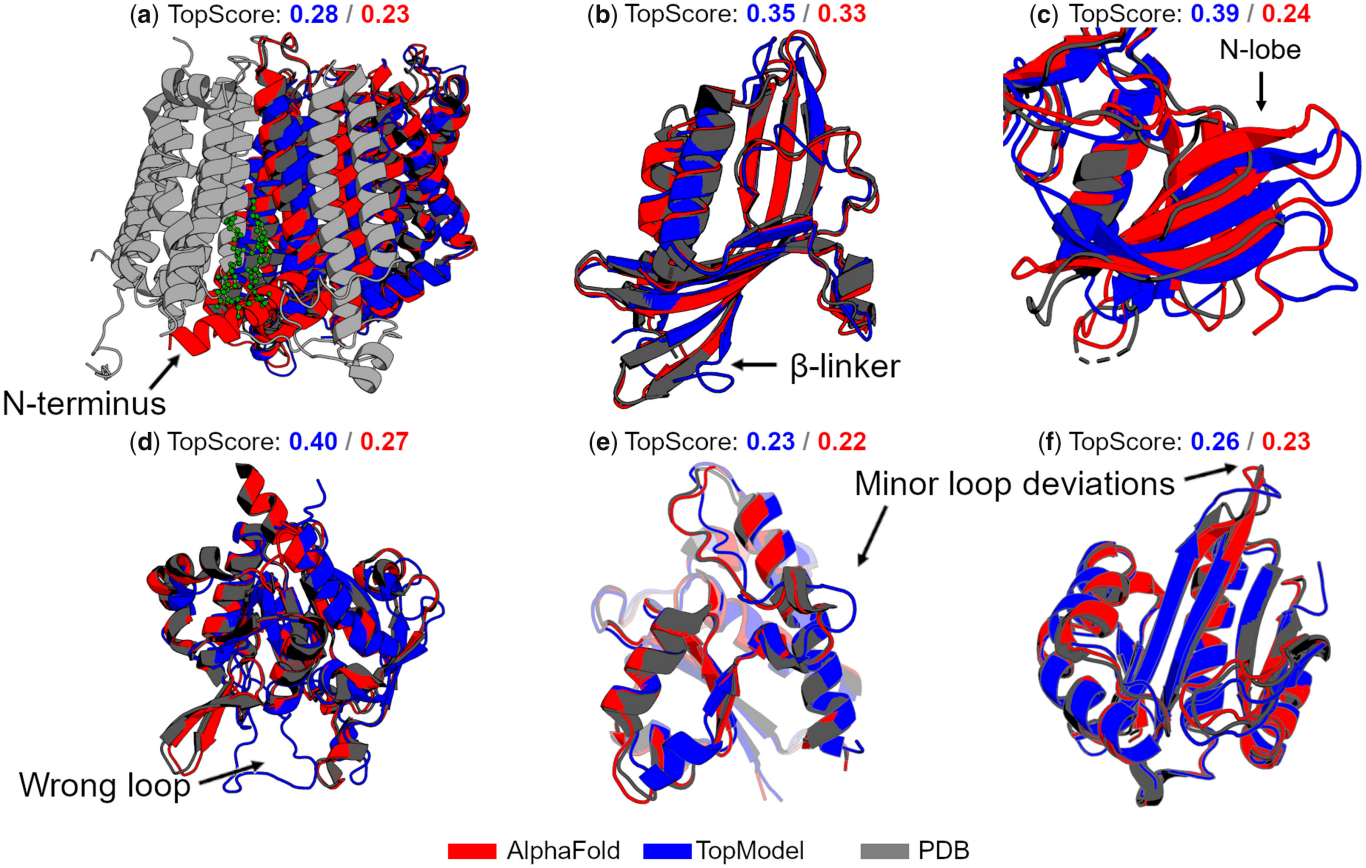
Comparison to experimental structures. An overview of six crystal structures (gray) obtained from the Protein Data Bank compared to AlphaFold2 (red) and TopModel (blue) models. Below the structures, the TopScore for the TopModel and AlphaFold2 models is shown. The arrows correspond to structural features discussed in the text. All structures have been deposited recently and are not present in the training databases for either method. (a) NADH-ubiquinone oxidoreductase (PDB ID 7A23) in the plant mitochondrial respiratory complex I. In the AlphaFold2 model, an α-helix incorrectly protrudes in the direction of the complementary subunit. (b) Salicylate 5-hydroxylase (PDB ID 7C8Z). Both methods produce a good-quality structure. (c) Yeast TFIIK (Kin28/Ccl1/Tfb3) complex (PDB ID 7KUE). Both methods predict a larger b-sheet region that is characterized as mostly coil in the PDB file. (d) Gamma-glutamyl-gamma-aminobutyrate hydrolase (PDB ID 6VTV). The TopModel model mispredicts part of the fold and creates a random coil region instead of a β-sheet. (e) Phosphotyrosine protein phosphatase 1 (PDB ID 7CUY). In the TopModel model, a small random coil region diverges from the PDB structure and AlphaFold2 model. (f) N-α-acetyltransferase 30 (PDB ID 7L1K). Both methods create a good-quality structure

Besides comparing both structure prediction methods, we also compare both methods to recently released X-ray crystallography structures in the PDB (Fig. 4). These structures are chosen such that they were not part of the training data for AlphaFold2 or TopModel. In general, both methods produce models of comparable quality, with AlphaFold2 models having a better average TopScore of 0.04.

In NADH-ubiquinone oxidoreductase (Fig. 4a), the membrane domain is modeled well by both methods, except for the N-terminus, which is uncharacterized in PDB ID 7A23. In the case of TopModel, this part is modeled as a disordered region, which gets removed by postprocessing. AlphaFold2 predicts this region as an α-helical structure, albeit with low confidence. In both cases, the N-termini stick straight through the binding site for cardiolipin in the crystal structure. This site is recognized as an important site for the stability of the protein domain (Soufari et al. 2020). In Salicylate 5-hydroxylase (Fig. 4b), two models were predicted with structural features of excellent quality. However, the loops between the β-strands and the random loops deviate from the crystal structure, which lowers the global score. For the Yeast TFIIK (Kin28/Ccl1/Tfb3) complex (Fig. 4c), we focus on the specific N-lobe region of the enzyme (van Eeuwen et al. 2021). The difference in TopScore values between both methods is due to the improved structural features in that domain of the AlphaFold model. However, both methods create a similar deviation in the N-lobe region in that they modeled a larger β-sheet. Note that the crystal structure (PDB ID 7KUE) was refined in conjunction with enzyme CDK2 (PDB ID 1FIN), which is present in both the training database for TopModel and AlphaFold2. If we compare the β-sheet region among the models and CDK2, the models agree perfectly with CDK2 (van Eeuwen et al. 2021), where this β-sheet region is much larger. Likely, both methods learned to model this section according to CDK2 instead of predicting the smaller β-sheet seen in Kin28. The TopModel model of Gamma-glutamyl-gamma-aminobutyrate hydrolase (Fig. 4d) is an example of insufficient quality. Even though most of the structural features are in good agreement, a loop region should have been modeled as a β-sheet. Finally, for both Phosphotyrosine protein phosphatase (Fig. 4e) and N-α-acetyltransferase 30 (Fig. 4f), the models generated by either method are very good. The TopScore is high, and the structural features are in excellent agreement with the PDB structures (PDB ID 7CUY and PDB ID 7L1K).

**Figure 5:**
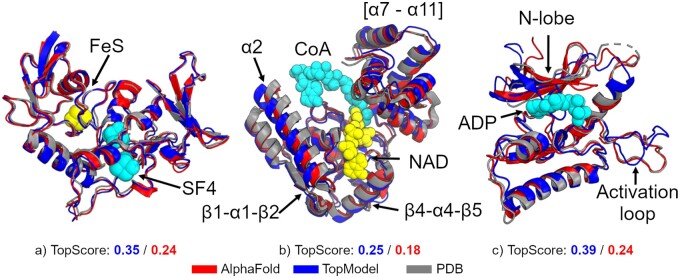
Comparison of binding sites. A cut-out of the binding site for three crystal structures (gray) obtained from the Protein Data Bank and compared to AlphaFold2 (red) and TopModel (blue) models with the corresponding binding ligands. The arrows correspond to structural features discussed in the text. All structures have been deposited recently and are not present in the training databases for either method. Only the structural features close to the binding sites are visualized to improve clarity. (a) The matrix arm of plant mitochondrial respiratory complex I (PDB ID 7A23). In both models, the structures around the FeS and SF4 ligands are of excellent quality. (b) Mycobacterium tuberculosis protein FadB2 (PDB ID 6HRD). Both methods model the two Rossman folds, β1-α1-β2 and β4-α4-β5, and the α7-α11 region very well. The TopModel slightly deviates in the α2 helix. (c) Yeast TFIIK (Kin28/Ccl1/Tfb3) complex (PDB ID 7KUE). AlphaFold2 predicts an excellent model, while TopModel places the N-lobe through the ADP binding region. This loop conformation represents another state found in the kinase structure template PDB ID 4ZSG in complex with an inhibitor

Furthermore, we take a detailed look at three binding interfaces for recently released X-ray crystallography structures in the PDB (Fig. 5). Only the structural features and loops close to the binding sites are visualized to improve clarity. In the matrix arm for plant mitochondrial respiratory complex I (PDB ID 7A23, Fig. 5a), FeS and SF4 clusters are important for the proton pump mechanism in ubiquinone reductase (Soufari et al. 2020; Parey et al. 2021). For both models, the structural details around the FeS and SF4 clusters are nearly identical to the crystal structure. In the Mycobacterium tuberculosis protein FadB2 (PDB ID 6HRD, Fig. 5b), the two Rossman folds, β1–α1–β2 and β4–α4–β5, and the α7–α11 region are very well modeled (Cox et al. 2019). The α2 helix in the TopModel model slightly points away from coenzyme A. Further investigation of the N-lobe in Yeast TFIIK reveals that for the TopModel model the flexible linker sticks into the ADP binding site and the activation loop deviates from crystal structure (Fig. 5c). Yet, predicting these residues exactly as in the crystal structure might be less crucial as they are shown to be the least stable residues of the enzyme (Luque and Freire 2000).

To conclude, both TopModel and AlphaFold2 can provide high-quality enzyme structural models with, in general, very good structural features of the binding sites. In some cases, TopModel falls short in modeling loops when compared to static crystal structures. However, the exact structure of these loop regions may often be less important due to the dynamic nature of proteins.

## 6 Conclusions

We have developed TopEnzyme, a database and framework for the structural coverage of functional enzyme space. By combining the TopEnzyme, SWISS-model repository, and AlphaFold DB databases, we provide the currently largest collection of enzyme structural models classified according to EC numbers in the UniProtKB/Swiss-Prot. TopEnzyme provides easy access to this collection with two methods: (i) A csv file containing all the metadata required for large-scale analyses. (ii) A treemap hosted on https://cpclab.uni-duesseldorf.de/topenzyme/ that allows the user to investigate specific enzyme functions.

With our in-house method TopModel, we added 9039 good-quality structural models, including 1297 ones of high quality. We compared a subset of these structures with AlphaFold2 models; on average, the TopScore between both models only differs by 0.04. Both methods can provide models with excellent structural features compared to experimental structures, although TopModel models sometimes differ in loop regions.

With this collection of enzyme structural models charted on functional space, researchers have access to a comprehensive and structured dataset, which should help to facilitate structure-guided investigations of specific enzymes and to develop predictive models for enzyme characteristics.

## Supplementary Material

btad116_Supplementary_Data

## Data Availability

TopModel models in .pdb format as a tar archive. Available at https://cpclab.uni-duesseldorf.de/topenzyme/ or http://dx.doi.org/10.25838/d5p-38. Csv file containing the meta-data for each UniprotAC identifier. Available at https://cpclab.uni-duesseldorf.de/topenzyme/ or http://dx.doi.org/10.25838/d5p-38.
